# Neuroprotection of retinal ganglion cells *in vivo* using the activation of the endogenous cannabinoid signaling system in mammalian eyes

**DOI:** 10.1042/NS20210038

**Published:** 2022-02-16

**Authors:** Greg Maguire, Christy Eubanks, George Ayoub

**Affiliations:** 1 California Physiological Society, Berkeley, CA, U.S.A.; 2 University of California, Department of Psychology, Santa Barbara, CA, U.S.A.; 3 Westmont College, Santa Barbara, CA, U.S.A.

**Keywords:** cannabinoids, glutamate, neurodegeneration, Retina

## Abstract

Cannabinoid and glutamatergic signaling systems in the human retina coexist and greatly influence one another. Under glaucomatous conditions, excess levels of glutamate accrete in the retinal ganglion cell (RGC) layer. The present study tests the putative neuroprotective effect mediated by cannabinoids at the CB1 and CB2 receptors. In the first experiment, mice were given intravitreal injections of 160 nmol N-methyl-d-aspartic acid (NMDA) in one eye and saline in the paired eye. In the second experiment, both eyes were given NMDA, while one of the two was additionally given the cannabinoid agonist WIN 55,212-2. Ten days later, animals were perfused and the retinae were dissected as wholemounts and stained with Cresyl Violet. Quantitative analysis revealed that 70% of the neurons in the retinal ganglion cell (RGC) layer exposed to NMDA underwent cell death. The addition of the cannabinoid CB1/CB2 agonist doubled the number of neurons surviving the NMDA treatment. These data provide evidence that cannabinoids, either exogenous or endogenous, may be harnessed to provide protection from neurodegenerative diseases, including glaucoma, and from glutamate-induced, and potentially other forms of neurotoxicity, under chronic or acute conditions.

## Introduction

Glaucoma is a disease of neurodegeneration of retinal ganglion cells (RGCs) [1]. The optic nerve comprises axons of these RGCs located primarily in the ganglion cell layer. Damage to these ganglion cells is observed clinically as increased cupping or excavation of the optic nerve [2]. Many factors are associated with increased risk of glaucoma, such as increased glutamate levels in human glaucomatous retina [3], visualized [4] as a cloud of glutamate over the RGC layer in experimental models of glaucoma [5], a greater ratio of inflammatory T cells [6] and an increased ratio of complement C3a to C3 [7], and the possibility of an age-associated down-regulation of the CB1 receptor coupled to G-protein α subtype i/o (G_i/o_) signaling system [8]. Activation of CB1 receptors has demonstrated efficacy in neuroprotection and a reduction in inflammation [9], and activation of CB2 receptors has been found to act broadly in many tissues to reduce the inflammatory immune responses [10,11]. Currently there is neither a proven direct treatment for the optic neuropathy of glaucoma, nor is there a clear understanding of the process that leads to these characteristic changes in the optic nerve.

In the past, treatment has focused on lowering intraocular pressure to reduce further damage to the optic nerve head [12]. However, as many as one-sixth of patients with glaucomatous damage show no evidence of elevated intraocular pressure [13]. For those with elevated pressure, even if the pressure is brought into the normal range, visual field loss and blindness continues to develop in 25–38% of patients [14]. These findings along with a host of others have made it evident that high intraocular pressure is only part of the process that leads to glaucomatous changes in the optic nerve [13–15]. This has led researchers to broaden their definition of both the disease process and possible treatments, including the use of molecules derived from mesenchymal stem cells to rescue degenerating neurons [16,17]. Importantly, both the CB2 signaling system [18] and mesenchymal stem cells [19] up-regulate heat shock protein (HSP)-mediated protection of cells from various forms of stress.

Glutamate is the most common excitatory neurotransmitter in the brain, including the retina, and toxic to neurons [20], including RGCs, leading to ganglion cell death [21,22]. Studies reveal that the glutamate concentration in the vitreous of glaucoma patients is twice the level of non-glaucomatous patients [3]. Experiments that chronically expose the retina to glutamate levels slightly higher than endogenous levels have demonstrated a glaucomatous degenerative pattern [23]. Numerous experimental studies have consequently attempted to develop neuroprotective agents to prevent the death of RGCs caused by toxic levels of glutamate [15,23,24]. One class of compounds that has generated considerable interest due to their potential for arresting glaucomatous neurodegeneration are the cannabinoids. Previous studies have revealed the presence of CB1 and CB2 receptors in various mammalian eyes, including the human retina [25–28]. Endogenous cannabinoids were found in the eye as part of an endogenous signaling system [29], and exogenous cannabinoids such as THC and various endogenous cannabinoids have been shown *in vitro* to be neuroprotective of cerebellar, hippocampal and neocortical neurons when challenged with toxic levels of glutamate [30–35]. The neuroprotective effects of cannabinoids on RGCs following glutamate toxicity have not been determined *in vitro* or *in vivo*. They have, however, been shown to reduce ganglion cell death following optic nerve crush [36], suggesting that they may be effective as a therapeutic agent. In the present study, we used an agonist that activates CB1 and CB2 to determine the effects of activating the endogenous cannabinoid signaling system in a retinal model of neurodegeneration, and demonstrate the ability of the synthetic cannabinoid CB1 and CB2 agonist WIN 55,212-2 to protect RGCs *in vivo* against NMDA excitotoxicity.

## Materials and methods

Experiments were performed on adult male and female C57/BL6 mice approximately 4 months of age, weighing 15–35 g. Mice were given food and water *ad libitum*. All studies were conducted at Westmont College and were approved by the Review Board in accordance with the principles and procedures outlined in the National Institute of Health (NIH) Guide for the Care and Use of Laboratory Animals.

### Chemicals

Three sterile solutions were administered by intravitreal injection: balanced saline solution (the Control condition), N-methyl-d-aspartic acid (the NMDA condition), and NMDA plus WIN 55,212-2 (the NMDA+WIN condition). WIN 55,212-2 is a CB1/CB2 receptor agonist [37,38]. The NMDA solution comprised 320 mM NMDA in balanced saline solution (net 160 nmol of NMDA were injected per eye). The WIN solution contained 0.5 mM WIN 55,212-2 in the NMDA solution using a DMSO vehicle (less than 0.1% of solution) to carry WIN (net of 0.25 nmol of WIN injected per eye). Both reagents were obtained from Sigma (St. Louis, MO).

### Intravitreal injections

Mice were anesthetized by intraperitoneal injection of 0.017 ml/g body weight of a solution containing 1.75% tribromoethanol and 1.75% tertiary amyl alcohol. A topical application of 0.5% proparacaine hydrochloride was administered prior to each intravitreal injection. A small incision was made with a 22-gauge needle in the dorsal limbus, through which a Hamilton syringe was then passed to administer the solution into the vitreal chamber. All eyes were injected with 0.5 µl of solution, performed under visual control using a binocular operating microscope. Occasionally this procedure produced cataracts (less than 5%) due to damage to the lens; these mice were not used in the analysis. In the first experiment, the right eye was injected with saline vehicle while the left eye was injected with NMDA. In the second experiment, the right eye was injected with NMDA and the left eye was injected with NMDA+WIN. For all injections, one dose was applied on day 1.

### Topical application of WIN55,212-2 to cornea

Previous studies have found WIN55,212-2 to affect brainstem activity when applied topically to the cornea [39] (Bereiter et al., 2002), and to diffuse systemically, including into the CNS, when applied as an IP injection [40]. We therefore topically applied WIN55,212-2 topically to one eye that had been treated with an intraocular injection of NMDA, and the other eye was served as a within subjects control using topically applied saline as the control in the NMDA injected contralateral eye.

### Tissue preparation

Ten days later, mice were sacrificed by an intraperitoneal injection of pentobarbital (120 mg/kg). Mice were perfused intracardially with 15 ml of 0.9% saline solution followed by 60 ml of 4% paraformaldehyde, and the eyes enucleated. The retinae were dissected and mounted on to gelatinized slides, ganglion cell layer up. Several radial cuts were made at the periphery, and the surface was cleaned and flattened with a fine brush. Each retina was immersed overnight in a solution containing 10% formaldehyde and 90% alcohol, being held flat under a coverslip and small weight. Retinas were subsequently hydrated and stained with 0.5–1% Cresyl Violet, dehydrated, cleared in hemo-de, and then mounted under coverslips.

### Morphometric analysis

The ganglion cell layer was imaged using a ×40 objective, and cells were counted within a square grid occupying 120 µm × 120 µm. Four such samples were taken at retinal loci midway between the optic nerve head and the retinal periphery in each quadrant of the flat-mounted retina. Individual cells intersecting the top and right edges of the grid were included in the counts, while cells intersecting the bottom and left edges were excluded. Quadrant counts were then averaged and corrected to obtain an average estimate of cell density for each eye, in cells per mm^2^. Morphologically distinguishable glial cells, endothelial cells, and immune cells were excluded from the cell count, but no attempt was made to distinguish between ganglion cells and displaced amacrine cells. Although RGCs predominate in the cells to be sampled, without specific staining methods for RBCs, we cannot rule out that cells other than RGCs were a part of our sample. Student’s one-tailed paired *t* tests were conducted to compare the effects of the treatments between the two eyes within each group, using a significance level of 0.05.

## Results

**Figure 1:**
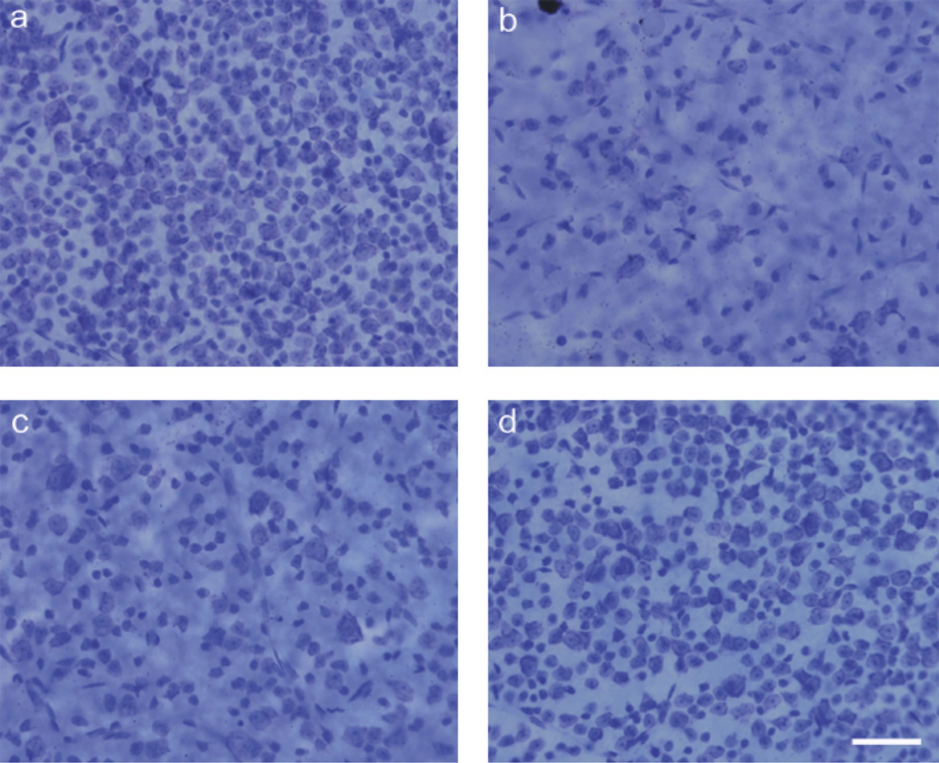
Photomicrographs of stained retinal ganglion cells from injected eyes Photomicrographs of mouse eyes from one mouse that had received: (**A**) an injection of saline, (**B**) an injection of NMDA. Photomicrographs of mouse eyes from one mouse that had received: (**C**) NMDA and (**D**) NMDA with WIN 55,212-2. The NMDA injections were found to significantly reduce the number of RGCs, and the addition of WIN 55,212-2 was found to protect against NMDA-induced neurodegeneration. Scale bar = 50 µm.

Figure 1 shows examples from pairs of eyes in each of the two experiments. The top pair of photomicrographs show the results from one mouse that had received an injection of saline and NMDA into the right and left eyes, respectively (experiment 1), while the bottom pair are images from a second mouse that had received an injection of NMDA (C) and NMDA + WIN 55,212-2(D) into the right and left eyes, respectively (experiment 2). A comparison of Figure 1A,B shows the conspicuous reduction in the density of neurons within the ganglion cell layer following NMDA treatment. Neurons of all sizes were affected, consistent with prior demonstrations in the literature that both ganglion cells and amacrine cells are excitotoxically ablated using this glutamate analog [23,41,42]. A comparison of Figure 1C,D shows that the coincident exposure of the NMDA-treated retina to WIN 55.212-2 ameliorates the excitotoxic damage produced by this glutamate analog.

**Figure 2:**
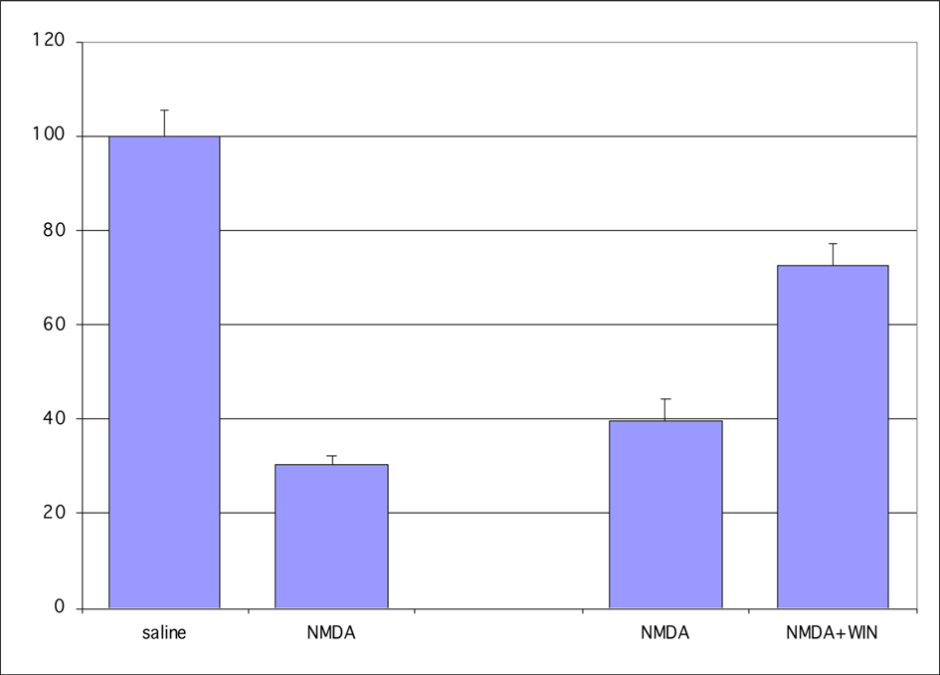
Percent of RGCs Saline served as the control (100%) against which NMDA was compared (30%). In the mice treated with NMDA versus NMDA+WIN 55,212-2, the mice injected with NMDA had ∼30% of their RGCs remaining, and the addition of WIN55,212-2 in the injection increased the remaining RGCs to ∼70%.

Figure 2 shows the quantification of these results. The saline-treated control eyes of nine mice in experiment 1 averaged 6288 cells per µm^2^, while paired eyes exposed to NMDA averaged 1898 cells per µm^2^. This difference is significant (*P*=0.0000003, SEM = 138, *t* test, *n*=9), being a 70% loss of cells during the 10-day treatment period. This compares well with the data of Li et al. (1999) [43], who found a 72.5% loss of RGCs using a similar NMDA dosage. In mice receiving NMDA injections with or without the putative neuroprotective agent WIN 55,212-2 (experiment 2), an average of 2504 cells per µm^2^ were seen in the NMDA-treated eyes, while an average of 4555 cells per µm^2^ were counted in the paired eyes receiving NMDA and WIN 55,212-2 simultaneously. This result was also significant (*P*=0.00002, SEM = 302, *t* test, *n*=15), indicating that a single application of WIN 55,212-2 was sufficient to nearly double the number of neurons surviving exposure to NMDA. Thus, while NMDA results in a substantial loss of neurons in the ganglion cell layer, the loss is reduced to only 28% following NMDA + WIN treatment.

In Figure 2, intraocular injection of WIN55,212-2 protects RGCs from NMDA-induced neurodegeneration. In the same mouse, control eye is taken as 100% and is the eye injected with saline (saline). The eye with NMDA injected into the eye alone (NMDA) has ∼30% of the RGCs surviving compared with saline control. In the same mouse, NMDA is injected into one eye (NMDA) and the contralateral eye is injected with NMDA and WIN55,212-2. In these mice, the NMDA injected eye has ∼35% of the RGCs surviving, whereas the contralateral eye injected with NMDA and WIN55, 212-2 has ∼65% of the RGCs surviving. The ordinate reflects normalized percentages compared with the saline control number of RGCs, which is 100%.

**Figure 3:**
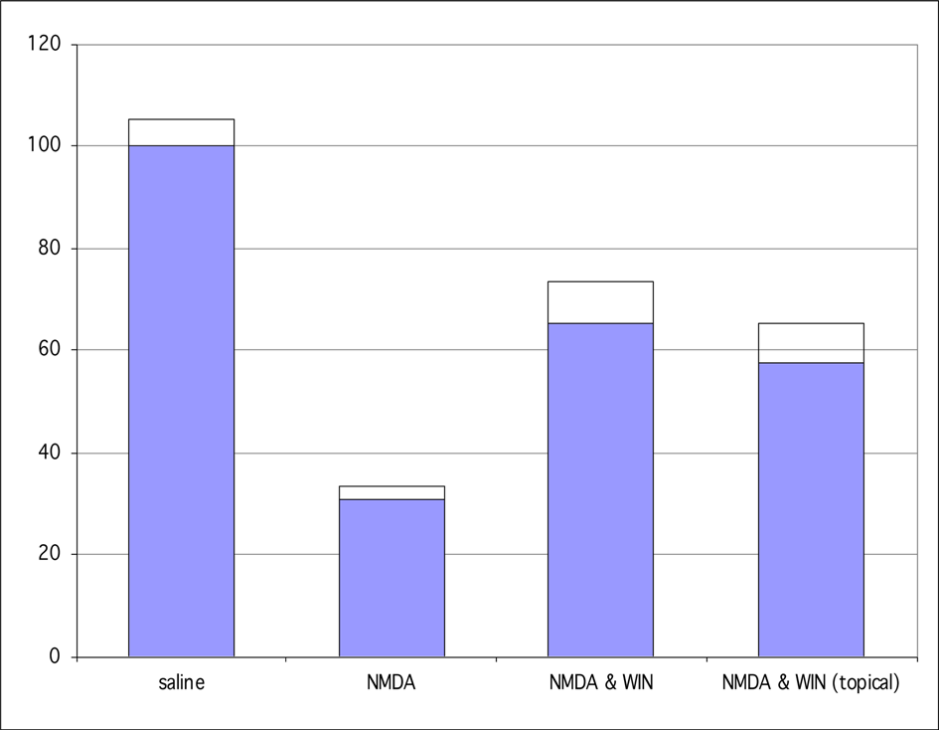
WIN55,212-2, a CB1/CB2 receptor agonist, topically applied to the cornea of one eye protects against NMDA-induced RGC neurodegeneration The ordinate reflects normalized percentages compared with the saline control number of RGCs which is 100%.

Figure 3 is a graphical display of the results obtained by topically treating the NMDA injected eyes with WIN55,212-2. In the control eyes, injected with NMDA and topically administered saline, the mean number of RGCs was 2922/µm^2^, and that for the NMDA-injected eye treated with topical WIN55,212-2 was 4088 µm^2^. These data were statistically significant (*P*=0.009, SEM = 355, *t* test, *n*=27). The higher value of RGC survival rate in the control eye of the mice administered topical WIN55,212-2 in the contralateral eye may reflect systemic crossover of the cannabinoid (Bereiter et al., 2002) from the contralateral eye that received topical WIN55,212-2.

**Figure 4:**
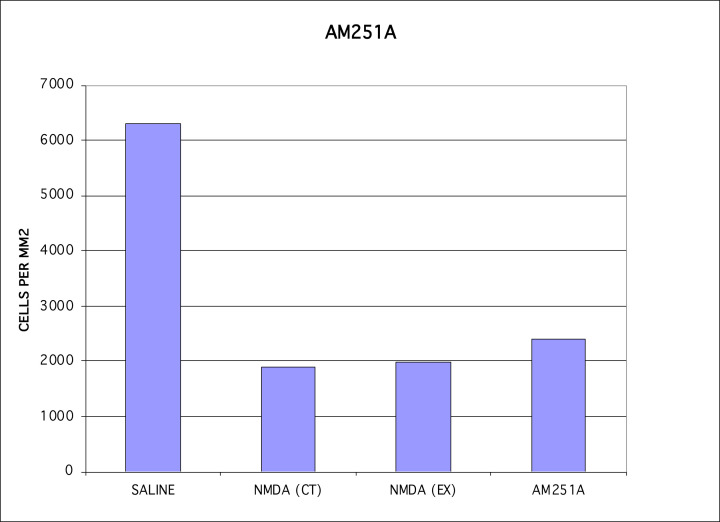
AM251A, a CB1 receptor antagonist, was used to block the effects of a CB1 receptor agonist The CB1 antagonist AM251A was able to largely block the CB1 agonist and allow NMDA-induced cytotoxicity of RGCs.

Next we used a CB1 receptor antagonist, AM251A [44], to determine whether antagonism of CB1 would prevent the CB1 receptor agonist, WIN55,212-2, from protecting against NMDA-induced cytotoxicity. We found a large reversal of the protection by the CB1 agonist of NMDA-induced cytotoxicity using an injection of AM251A that reached a final concentration of 10 nm in the eye (Figure 4). The lack of a full effect using the CB1 antagonist may reflect limited protection through activation of the CB2 receptor pathway, or insufficient dosing to fully act as an antagonist at CB1, and/or, because AM251A is light sensitive, that may have resulted in some light-induced degradation of AM251A in the eye compartment.

## Discussion

**Figure 5:**
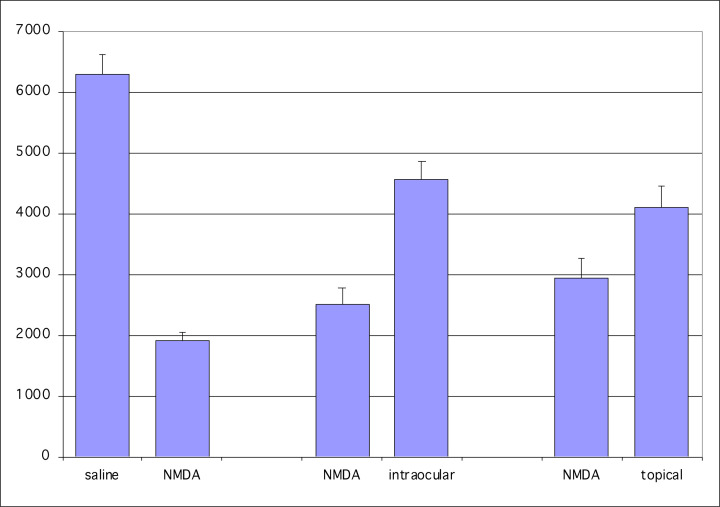
A comparison of the effects of WIN55,212-2 applied as a topical to the cornea (topical), injected intraocular (intraocular) and without WIN55,212-2 as a control (saline) The ordinate reflects mean cell number, and error bars are for the SEM.

An endogenous cannabinoid signaling system is conserved in vertebrate retinas, including human [26,27,29], and modulates glutamatergic activity in the retina, such that glutamatergic activity is reduced in a number of pathways [27,45,46]. The Maguire lab demonstrated an endogenous cannabinoid signaling system in vertebrate retinas replete with CB1 receptor localization and function [26,27], and then later reported for the first time that CB2 RNA was present in mammalian retina but were unable to identify the CB2 receptor [29]. The RNA data for CB2 were suggestive of a CB2 signaling system, but inconclusive without identification and localization of the receptor itself. Nonspecific binding of antibodies without proper controls can often result in false-positive hits for CB2 receptors, and this has likely plagued many of the studies in search for the CB2 receptor in central nervous system [47], of which the retina is a part. In 2018, the Straiker Lab [48], in a careful study employing a number of methods, including five commercial CB2 antibodies and a genetically modified mouse with an inserted eGFP reporter for the same bicistronic mRNA as the CB_2_ receptor [49], provided evidence that the CB2 receptor has a small baseline expression in normal retinas, but that CB2 receptor is up-regulated in the retina under pathological conditions, consistent with CB2 having its predominant role in immune function [50]. Indeed, evidence suggests that CB2 is up-regulated by inflammatory states [51]. Cannabinoids acting at CB1 receptors coupled to G_i/o_ have been found to be neuroprotective [52]. Although pressure control has been the major tool in the management of glaucoma, the strategy has poor efficacy and a better approach to preservation of the optic nerve more directly may be to use neuroprotective agents. In this study, WIN 55,212-2 was found to be neuroprotective, having elicited a decrease in NMDA-induced RGC death. The 58% rescue rate for RGCs compares well with the rescue rates in a study by Klocker et al. [53] where brain-derived neurotrophic factor was found to have a 27% rescue rate alone and a 68% rescue rate when combined with N-tert-butyl-(2-sulfophenyl)-nitrone. See (Figure 5) for a comparison of rescue by cannabinoids applied topically compared to intraocular injection.

Our *in vivo* test of a cannabinoid agonist to protect against glutamate excitotoxicity correlates with several *in vitro* studies on the efficacy of these agents. These studies all found that cannabinoids provided neuroprotection of cultured cortical, cerebellar, and hippocampal neurons [30–35]. The cannabinoid agonist WIN 55,212-2 has been found to bind the CB1 and CB2 receptors [54] to elicit the presently demonstrated neuroprotective effect [34]. Some studies have demonstrated neuroprotective actions by cannabinoids independent of CB1 or CB2 receptors [31,34], suggesting a novel receptor. Others have proposed that the cannabinoid protection results from decreasing the release of glutamate presynaptically [35]. Additional possibilities include permeating the membrane, blocking calcium channels, or acting as an antioxidant [30–35]. The neuroprotective effect of exogenous endocannabinoids in the retina may also be partially mediated through Muller glia [55]. Muller glial cells are one of a number of stem cells types found in retina. Like adult stem cells in other parts of the body, including adipose-derived mesenchymal stem cells [19,56], Muller cells exert their protective effects partially through resolving destructive proinflammatory signaling. Multiple lines of evidence suggests that Müller cells are dormant stem-like cells found throughout the retina and where they are a source of progenitor cells that can regenerate retinal neurons after injury [57]. Ciliary epithelia-derived cells, retinal pigment epithelium (RPE), and bone marrow stem cells (BMSCs) have also been found to be potential sources of progenitor cells that can migrate to the injured retina [58]. Endocannabinoids act tonically at the CB1 receptor to maintain survival of some stem cells [59], act to increase their metabolic viability by inducing HO-1-independent autophagy [60], and to increase their release of growth factors [61]. The mechanisms by which cannabinoid-induced neuroprotection occurs may prove to be a combination of all these mechanisms. Regardless of the mechanism, the present *in vivo* study suggests that cannabinoids possess great therapeutic potential for slowing the neurodegenerative process of glaucoma and other neurodegenerative disorders that involve excitotoxicity.

For a substance to have a role as a neuroprotective agent in glaucoma, it would ideally be delivered topically to the eye and usable repeatedly [15]. Minimal effects on vision and cognitive function are also desirable. Cécyre et al. [62] measured a change in oscillatory potentials of the ERG, reflecting retinal amacrine neuron function [63], and visual acuity using an optokinetic response in mice with CB2 activation. Past experiments have revealed the pressure-lowering effects of cannabinoids upon topical application [64,65] indicating their ability to penetrate the eye. Pinar-Sueiro et al. [66] have found that topically applied WIN55,212-2 is efficacious in reducing RGC loss in an increased IOP model of glaucoma in mice. We are currently testing the neuroprotective effects of other cannabinoids when topically applied.

Subtleties in the neuroprotective effects observed in our study will require further work. As an example, the neuroprotective actions of WIN55,212-2 at the CB2 receptor may occur only at the onset of neuroinflammation, and not in later phases of the neurodegenerative sequalae [67]. And WIN55,212-2 has been suggested to quell reactive astrocyte inflammation through a noncanonical pathway [68]. Thus, WIN55,212-2 may act in time and space through myriad pathways, such as direct actions at CB2 receptors on RGCs [29,49], and indirect actions of CB1 at many sites in the retina [28,29], to achieve the protection demonstrated in our study.

Neurodegenerative diseases increase with age as the cannabinoid signaling systems in brain decline [69]. For example, during normal aging the level of the major endogenous cannabinoid, 2-arachidonoylglycerol and the coupling of the CB1 receptors to G_i_ protein are significantly reduced [70,71]. Data from Bilkei-Gorzo et al. [69] suggest that the age-related decline in cannabinoid signaling may be responsible or at least contribute to the development of astrocyte aging. Given that increased age, and associated non-genetic factors, increase the risk for neurodegenerative diseases, including glaucoma, methodologies to renormalize the physiology of aged individuals may be an important strategy [72]. Thus returning the physiology to a normal state once the neurodegeneration has begun, may serve to remediate the damage as was found in an *in vitro* model of neurodegeneration [17]. The Maguire et al. study [17] found that returning the normal set of molecules released from stem cells that maintain neural proteostasis, can remediate key biomarkers of neural degeneration, including neurite outgrowth and stress granuoles. Likewise, when considering the endogenous cannabinoid system of the nervous system, returning the cannabinoid signaling system to a normal state may be an important strategy in preventing and remediating neurodegenerative diseases. Using a systems therapeutic approach [73], molecules that modulate both the cannabinoid system and the neural stem cell system may work collectively in a synergistic manner to better prevent and remediate disease of the nervous system. Renormalizing the aged CB1 and CB2 receptor signaling systems activity may therefore be one, reductionist part of physiological renormalization strategy involving a systems approach where many pathways are renormalized. For example, when neural stem cells differentiate into neurons, HSP function in the neurons is lost or reduced [74] and a loss of proteostasis [75] may occur in the neuron if surrounding neural stem cells no longer shuttle HSPs to the neuron [17]. Therefore, the induction of HSP activity by CB2 [18,76], and the addition of HSP by the delivery of mesenchymal stem cell exosomes [77], in concert, along with the pleiotropic effects of CB1 and CB2 on the glutamatergic and other pathways, may synergistically renormalize physiology through a systems therapeutic approach, serving to better prevent and remediate neurodegenerative diseases.

## Conclusions

In summary, we have demonstrated that the cannabimimetic drug, the CB1 and CB2 receptor agonist WIN55,212-2, acts to protect RGCs from NMDA-induced excitotoxicity in an *in vivo* mouse model. This further indicates the potential for therapeutic applications of cannabinoids in neurodegenerative diseases, including glaucoma.

## Data Availability

Data are available on request.

## Abbreviations

G_i/o_: G-protein α subtype i/o
HSP: heat shock protein
NMDA: N-methyl-d-aspartic acid
RGC: retinal ganglion cell
